# Triborheological Analysis of Reconstituted Gastrointestinal Mucus/Chitosan:TPP Nanoparticles System to Study Mucoadhesion Phenomenon under Different pH Conditions

**DOI:** 10.3390/polym14224978

**Published:** 2022-11-17

**Authors:** Gustavo Ruiz-Pulido, David Quintanar-Guerrero, Luis Eduardo Serrano-Mora, Dora I. Medina

**Affiliations:** 1 Tecnologico de Monterrey, School of Engineering and Sciences, Atizapan de Zaragoza 52926, Estado de México, Mexico; 2 Laboratorio de Investigación y Posgrado en Tecnología Farmacéutica, FES-Cuautitlán, Universidad Nacional Autónoma de México, Cuautitlán Izcalli 54745, Estado de México, Mexico; 3 Tecnologico de Monterrey, Institute of Advanced Materials for Sustainable Manufacturing, Monterrey 64849, Nuevo Leon, Mexico

**Keywords:** rheological synergism, mucoadhesion, reconstituted mucus, chitosan:TPP nanoparticles, gastrointestinal pH

## Abstract

Polymeric nanoparticles have attracted much attention as pharmaceutical delivery vehicles to prolong residence time and enhance the bioavailability of therapeutic molecules through the mucoadhesive phenomenon. In this study, chitosan:TPP nanoparticles were synthetized using the ionic gelation technique to analyze their mucoadhesive interaction with reconstituted porcine gastrointestinal mucus from a triborheological point of view under different pH conditions (pH = 2.0, 4.0, 6.0 and 7.0). The triborheological profile of the reconstituted mucus was evaluated at different pH environments through the oscillation frequency and the flow sweep tests, demonstrating that the reconstituted mucus exhibits shear thinning behavior regardless of pH, while its viscoelastic properties showed a change in behavior from a polymeric solution performance under neutral pH conditions to a viscoelastic gel under acidic conditions. Additionally, a rheological synergism analysis was performed to visualize the changes that occur in the viscoelastic properties, the viscosity and the coefficient of friction of the reconstituted mucus samples as a consequence of the interaction with the chitosan:TPP nanoparticles to determine or to discard the presence of the mucoadhesion phenomenon under the different pH values. Mucoadhesiveness evaluation revealed that chitosan:TPP exhibited strong mucoadhesion under highly acidic pH conditions, below its pKa value of 6.5. In contrast, at neutral conditions or close to its pKa value, the chitosan:TPP nanoparticles’ mucoadhesiveness was negligible.

## 1. Introduction

Mucus is a viscoelastic gel that protects the wet epithelial surfaces of gastrointestinal tissues [1] through several essential functions, such as: preventing the entry of pathogens and harmful molecules into the body, maintaining proper hydration of the tract by limiting the loss of water, lubricating the gastrointestinal tract to reduce the mechanical stress produced by swallowing and digesting food and proving a selective absorption of elemental molecules (nutrients) and gases (oxygen and carbon dioxide) [2,3,4,5].

On the other hand, gastrointestinal mucus consists of 95% of water, with the remaining 5% forming different molecules, including glycoproteins, antibodies, lipid complexes, inorganic ions, growth factors, DNA, cells and mucin [6,7]. Mucin, the main component of the mucus, is formed by a family of high molecular weight (10–40 MDa) gel-forming glycosylated proteins secreted by goblet cells and submucosal glands from the apical epithelium [8]. 

Mucin consists of a protein core with regions rich in serine and threonine, enabling a high grade of O-glycosylation that makes it resistant to enzymatic degradation and capable of holding water, giving it its hydrogel properties [7]. Moreover, mucins can interact covalently (disulfide bonds) and non-covalently (electrostatic and hydrophobic interactions) with different mucus components (lipids, proteins, salts, cellular debris, among others) or another mucin molecule to form a unique viscoelastic gel layer [9] that is intimately correlated with the porosity of the mucus structure and its rheological properties [10]. The high number of oligosaccharide chains present in mucin molecules provides a negative charge to the mucus due to the carboxyl and sulfate groups [2]. 

In the same way, the wide range of pH variation (between 1.0 and 8.0) throughout the gastrointestinal tract, considering from the oral cavity to the colon, represents a fundamental variable in the study of gastrointestinal mucus rheology. Since the environmental pH plays a key role in the structural and conformational modifications that mucus undergoes and, consequently, in the variations of its rheological properties [11]. Mainly in the highly acidic environment of the stomach that promotes the solid–gel transition of mucus as a fundamental role in protecting stomach epithelium from gastric acids during active digestion [12]. 

By considering the fluctuating pH conditions in the gastrointestinal tract, different polymer-based approaches have been studied for drug delivery purposes, including nanoparticles [13], hydrogels [14], nano-doped composites [15], nanofibers [16] and nanogels [17]. Nevertheless, nanoparticles represent the most studied polymeric nanocarrier in the pharmaceutical industry [18]. Nanoparticles are spherical colloidal structures with an approximate size of 10 to 1000 nm in diameter, which can be prepared with natural, synthetic or a mixture of biocompatible polymers [19,20]. Polymeric nanoparticles are commonly used for drug delivery purposes based on their high encapsulation efficiency, ability to control bioavailability, lifetime and release time of the molecules inside the body [21,22]. Moreover, its small size could facilitate pass across the mucus barrier, as long as they do not present strong hydrophobic, electrostatic or hydrogen bond interactions with the mucin [1].

Chitosan is a natural cationic polymer synthetized by the N-deacetylation of chitin. This biopolymer exhibits good biodegradability, high stability, low toxicity, biocompatibility, mucoadhesiveness, sustained release and tuneable physical properties [23,24,25,26]. However, chitosan exhibits pH-responsive properties due to the presence of several amino groups in its structure, which are protonated in acidic conditions [27,28]. However, when the amino groups are deprotonated at neutral or alkaline pH, chitosan becomes insoluble, affecting its potential uses [29]. Thus, different alternatives were studied to enhance its drug delivery properties, for instance, the addition of an alkyl [30], carboxyl [31] or sulfate [32] group to increase chitosan solubility [33]. 

Mucoadhesion refers to the attachment of a natural or synthetic molecule to mucus for extended periods of time through different interfacial forces [34]. The mucoadhesive properties of nanomaterials have attracted great interest among scientists, so the evaluation of mucoadhesive properties is quite relevant. Rheological synergism has emerged as an indirect method to evaluate the adhesion strength of the interfacial layer formed among mucin and polymeric nanoparticles [35,36]. This method allows predicting the mucoadhesive profile of a polymer by measuring the synergy generated in the dynamic modules (*G*′ and *G*′′) or in the viscosity (η) when the interpenetration of polymer and mucin chains occurs during the phenomenon of mucoadhesion [37,38].

This work characterizes the viscoelastic, viscous and lubricating properties of reconstituted mucus through the fluctuating pH conditions to which mucus is subjected throughout the gastrointestinal tract with the aim of analyzing how mucus physicochemical changes affect its biological properties. Considering that mucus undergoes a pH-dependent transition when the gastrointestinal conditions change from acidic to neutral pH. However, this research focuses mainly on the application of the rheological synergism method to study the presence of the mucoadhesive phenomenon with the objective of determining the acidic conditions within the gastrointestinal tract that promote a greater degree of adhesion between mucus and chitosan:TPP nanoparticles. Additionally, this study analyzes the implications of the mucoadhesive phenomenon in the lubricating properties of mucus under different acidity environments.

## 2. Materials and Methods

### 2.1. Materials

Mucin from porcine stomach type III, bound sialic acid 0.5–1.5%, partially purified powder and Chitosan, low molecular weight, were purchased from Sigma-Aldrich (Toluca, México). Simulated Intestinal Fluid TS was obtained from RICCA Chemical. Sodium Tripolyphosphate (TPP) was acquired from Merck. All the other reagents were of analytical grade. 

### 2.2. Reconstituted Mucus Preparation

The preparation and manipulation of the reconstituted mucus samples were carried out under sterile conditions. Each sample of reconstituted mucus was prepared by diluting 500 mg of mucin in 10 mL of simulated intestinal fluid (SIF) to obtain solutions with a final mucin concentration of 5% (*w*/*v*). The solutions were incubated at 37 °C under constant agitation at 200 rpm for 24 h. At the end of the incubation period, each sample was carefully checked to ensure that it maintained its original pale yellow color and a completely homogeneous consistency to ensure that there was no contamination. Subsequently, the pH of each solution was adjusted to the corresponding value (2.0, 4.0, 6.0 or 7.0) to perform all the different analyses. The pH adjustment was made by adding the appropriate volumes of 1 M HCl or 1 M NaOH until the expected pH was obtained.

### 2.3. Synthesis of Chitosan:TPP Nanoparticles by Ionic Gelation Method

Initially, two solutions were prepared to perform the ionic gelation method for the synthesis of chitosan:TPP nanoparticles. A 3 mg/mL solution of chitosan was prepared by adding 300 mg of chitosan into 100 mL of diluted acetic acid (1%). Simultaneously, a 1 mg/mL solution of TPP was prepared by adding 50 mg of TPP into 50 mL of 1% acetic acid solution. The pH of both solutions was adjusted to 5.0 using 1 M HCL. The chitosan solution was stirred at 800 rpm for 60 min, while the TPP solution was stirred for 30 min. Next, TPP solution was added dropwise to the chitosan solution, which was under magnetic stirring at 800 rpm. The mixture was stirred for 60 min at 800 rpm. Then, the mixture was sonicated for 30 min at 40 kHz. Finally, the nanoparticle solution was frozen at −20 °C and placed inside the freeze dryer for 48 h. 

It is emphasized that before some characterization methodologies (size, polydispersity index and zeta potential measurements) and triborheological tests (including rheological synergism), the lyophilized nanoparticles were diluted in distilled water at 3 mg/mL concentration and sonicated for 20 min at 40 kHz. Then, the pH of each solution of nanoparticles was adjusted to 2.0, 4.0, 6.0 or 7.0, respectively. 

### 2.4. Chitosan:TPP Nanoparticles Characterization

#### 2.4.1. Particle Size, Polydispersity Index and Zeta Potential Measurement

Mean hydrodynamic diameter, polydispersity index (PDI) and zeta potential of chitosan:TPP nanoparticles were measured at 25 °C with 2 min of equilibrium using a Malvern Zetasizer Nano ZS. Dynamic light scattering (DLS) was used for the measurements of particle size and PDI with a laser wavelength of 633 nm and a 173° detection angle inside DTS0012 disposable cuvettes. Moreover, for zeta potential measurements, the electrophoretic light scattering (ELS) technique was utilized with DTS1070 disposable cuvettes. 

#### 2.4.2. Morphology

The analysis of chitosan:TPP nanoparticles morphology was performed by a JEOL JSM 6360 scanning electron microscope operated at an applied voltage of 15 kV using a high vacuum of the beam diameter and secondary electrons at magnifications of 5000×. Before the analysis, freeze-dried samples were placed on the sample holder and gold-sputtered on a JEOL JFC-1100E to enhance their conductivity properties.

Additionally, lyophilized nanoparticles were diluted in distilled water at a concentration of 3 mg/mL and sonicated for 20 min at 40 kHz. A drop of the solution of nanoparticles was placed on the sample holder and dried at room temperature for 48 h under a vacuum. The dried sample was gold sputtered and analyzed in the SEM at an applied voltage of 20 kV using a high vacuum of the beam diameter and secondary electrons at magnifications of 1600×. 

### 2.5. Rheology of Reconstituted Mucus

Rheological profiling of reconstituted mucus was performed with a TA Instruments Hybrid Rheometer DHR-3 using a cone-and-plate configuration formed by a 60 mm diameter stainless steel cone geometry (0.9969°) and a Peltier system to control the sample temperature at 37 °C to mimic human body conditions. The analysis of the rheological properties of reconstituted mucus under different pH values included oscillation frequency and flow sweep tests. The oscillation frequency test was performed with an angular frequency range between 0.1 and 100 rad/s at a strain of 1% to identify reconstituted mucus storage (*G*′) and loss (*G*′′) modulus. While the flow sweep test was carried out using shear rate values between 0.1 and 1000 s^−1^ to determine reconstituted mucus viscosity. 

### 2.6. Rheological Synergism

The rheological tests described in Section 2.5 were repeated using the solutions of chitosan:TPP nanoparticles and mixtures of reconstituted mucus with chitosan:TPP nanoparticles as samples at the different pH values studied (2.0, 4.0, 6.0 or 7.0). The obtained results were compared with reconstituted mucus a) viscoelastic and b) viscous profiles to determine the presence of mucoadhesion phenomenon between reconstituted mucus and chitosan:TPP nanoparticles:

(a) The values of the elastic modulus (*G*′) were compared to evaluate the mucoadhesive interactions among reconstituted mucus and chitosan:TPP nanoparticles. The evaluation was calculated as Δ*G*′ using Equation (1):
  (1) $$\Delta \;G^{\prime }={G^{\prime }}_{mix}-({G^{\prime }}_{muc}-{G^{\prime }}_{np})$$
  where Δ*G*′ refers to the mucoadhesive interaction term (the elastic component is interpreted as the interaction between nanoparticles and mucus), *G*′*_mix_* refers to the elastic modulus of the mixture, *G*′*_muc_* refers to the elastic modulus of the reconstituted mucus and *G*′*_np_* refers to the elastic modulus of the chitosan:TPP nanoparticles;
(b) The viscosities of the three samples (mucus, nanoparticles and mixture) were contrasted to evaluate mucoadhesion through Equation (2):
  (2) $$\Delta \;\eta^{\prime }={\eta^{\prime }}_{mix}-({\eta^{\prime }}_{muc}-{\eta^{\prime }}_{np})$$
  where Δ*η*’ refers to the viscosity due to mucoadhesive interaction, *η*’*_mix_* refers to the viscosity of the mixture, *η*’*_muc_* refers to the viscosity of the reconstituted mucus and *η*’*_np_* refers to the viscosity of the chitosan:TPP nanoparticles.

### 2.7. Triborheology of Reconstituted Mucus

Triborheological profiles of reconstituted mucus under different pH conditions were evaluated in the DHR-3 Hybrid Rheometer (TA Instruments) using a plate-and-plate configuration conformed by a 40 mm diameter stainless steel plate and a Peltier system that acts as a temperature controller (37 °C) during the tests. The coefficient of friction was evaluated by performing a flow sweep test from 0.1 to 50 rad/s, applying a normal force of 1 N. 

Additionally, the triborheological performance of the mixture (reconstituted mucus–chitosan:TPP nanoparticles) was measured under the same conditions and compared with reconstituted mucus performance to evaluate the alterations in the coefficient of friction produced by the mucoadhesive interaction. 

### 2.8. Statistical Analysis

All experiments were performed in triplicate, and the data shown are expressed as mean ± standard deviation. One-way analysis of variance (ANOVA) and post hoc Tukey tests was calculated with a significant level of *p* < 0.05 using Minitab 19 software (Microsoft, Redmond, WA, USA).

## 3. Results and Discussions

### 3.1. Triborheological Profiling of Reconstituted Mucus under Gastrointestinal pH Variations

Triborheology tests were carried out to determine the variations in the viscoelastic, viscous and lubricant profiles that reconstituted mucus undergoes through the different pH conditions presented in the gastrointestinal tract (pH = 2.0, 4.0, 6.0 and 7.0), considering a temperature of 37 °C to mimic the human body.

**Figure 1:**
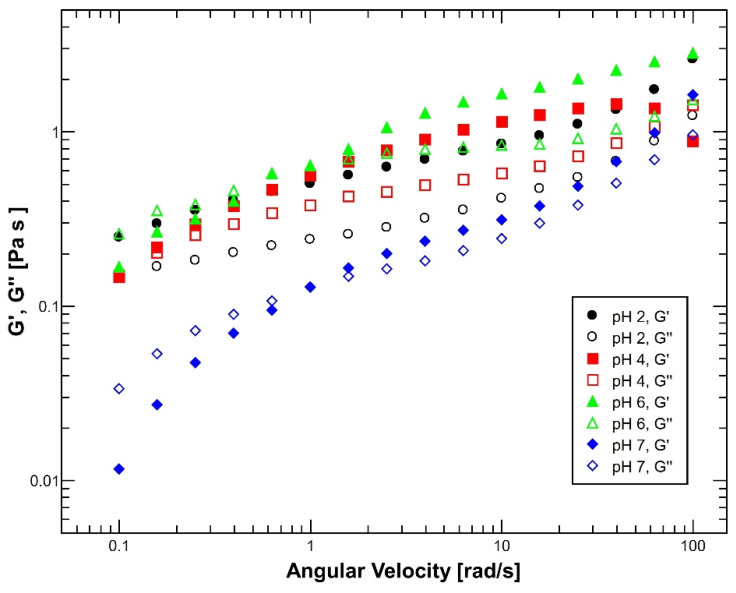
Evaluation of the viscoelastic behavior of reconstituted mucus at 37 °C under different pH conditions. At pH 2 (black dots), mucus exhibited an elastic behavior with a dominant elastic modulus (*G*′ > *G*′′). Similarly, at pH 4 (red squares), mucus kept an elastic behavior (*G*′ < *G*′′) but with less separation among the modulus and a crossover at high angular velocity (~82 rad/s). Instead, at pH 6 (green triangles) and pH 7 (blue diamonds), mucus showed an initial viscous behavior (*G*′′ > *G*′) until a crosslink around 1–1.5 rad/s for a characteristic polymeric solution performance. Emphasizing that at pH 7, mucus exhibited a significant decrease in its modulus. N = 5.

Oscillation frequency tests showed that reconstituted mucus exhibited significant changes in its viscoelastic properties depending on the pH conditions, as Figure 1 shows. All of these viscoelastic changes are intimately related to the pH-dependent structural changes that mucin undergoes [39]. Moreover, the tests were performed in the angular velocity range from 0.1 to 100 rad/s with the purpose of analyzing the linear viscoelastic region (LVR), which corresponds to values where the structure is stable to reliably evaluate its viscoelastic performance [40].

At highly acidic conditions (pH = 2), the viscoelastic behavior exhibited a clearly gel-like response since the storage modulus (*G*′) is predominant over the loss modulus (*G*′′) all the time. This behavior is explained by the protonation of the carboxylic groups and the breaking of disulfide groups of the mucin, which induces an unfolding and exposure of hydrophobic regions [11]. Moreover, the electrostatic repulsive forces among negatively charged sialic acid residues induce an extended conformational structure [41]. Thus, the mucin tertiary structure acquires a rodlike confirmation by complex interactions between the hydrophilic and hydrophobic regions of the glycoproteins (including the protein core), stimulating the formation of non-covalent crosslinking that favors the stabilization of water molecules by mucin hydrophilic groups, forming a protective gel [42,43] with the objective of restricting the passage of gastric juices and prevents stomach self-digestion by its own acids due to the highly acidic environment of the stomach [12,44].

Instead, under neutral pH conditions (pH = 6 and 7), mucus behaves as a viscous polymeric solution with an initial dominance of the loss modulus (*G*”) at very low frequencies followed by a modulus crossover to storage modulus (*G*′) dominance around an angular frequency of 1 rad/s. This performance is typical in solutions that act as weak gels [45]. In this case, the behavior is produced as a result of the different electrostatic interactions that are established among polysaccharide side chains and disulfide bonds (S-S) at the cysteine terminals of mucin molecules, improving the entanglement of the chains and leading to a random coil conformation of mucin molecules that results in enhanced viscosity [40,46,47,48]. 

Moreover, under slightly acidic conditions (pH = 4), the transition from a weak gel behavior to a polymeric solution can be visualized because the gap between storage (*G*′) and loss (*G*′′) modulus is significantly reduced. Additionally, the order of the storage modulus is reduced by half, while the order of magnitude in the viscous modulus is lightly increased. The situation was expected, considering that the mucus isoelectric point is found around the pH range of 2.0–3.0, which represent the critical point in the charge shift and conformational structure of mucus [43,49].

**Figure 2:**
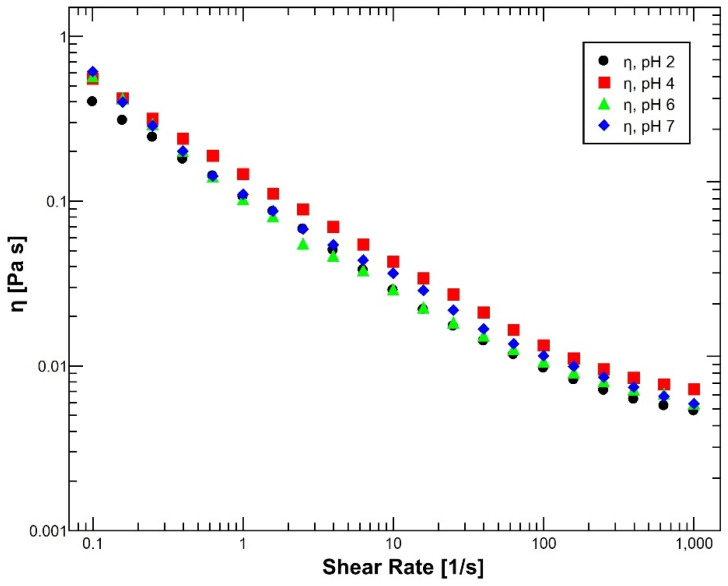
Evaluation of the viscous behavior of reconstituted mucus at 37 °C under different pH conditions: pH 2 (black dots), pH 4 (red squares), pH 6 (green triangles) and pH 7 (blue diamonds). Regardless of the pH, apparent viscosity (η) exhibited similar performances with a negative slope showing characteristic shear thinning properties. N = 5.

In the same way, flow sweep studies of reconstituted mucus confirmed that regardless of the pH conditions, mucus preserves its typical non-Newtonian shear-thinning nature, with a reduction in the viscosity values of the different samples as the shear rate increased during the application of a constant shear flow, as Figure 2 shows. This performance is essential for the normal function of the gastrointestinal mucus, which constitutes an unstirred aqueous layer that provides lubrication and protection against shearing forces and foreign pathogens [50,51]. However, the variation in the viscosity values among the different pH environments is minimum and not clearly seen due to the use of partially purified gastric mucin, which results in reduced physical crosslinks in mucus as a result of the loss of some components during the purification process [43]. Highlighting that the highest viscosity values were achieved at pH 4 when mucus undergoes a conformational transition from a rodlike conformation (elastic behavior) to a random coil conformation (viscous behavior). 

**Figure 3:**
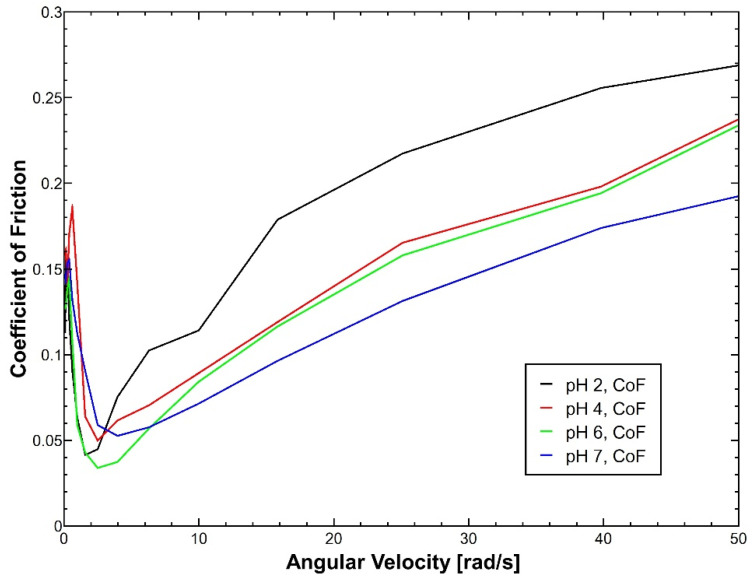
Stribeck curve to evaluate the coefficient of friction (CoF) of reconstituted mucus at 37 °C under an applied pressure of 1 N and different pH conditions: pH 2 (black), pH 4 (red), pH 6 (green) and pH 7 (blue). Independently of the pH, all the samples exhibited a similar performance during the boundary and mixed lubrication regimes. Instead, during the hydrodynamic regime, the more acidic sample showed a critical increase in its CoF in comparison with the other samples as a result of the conformational change that mucus undergoes around its isoelectric point (inside the pH range 2.0–3.0). N = 5.

Additionally, the lubricant performance of reconstituted mucus was tested under an applied pressure of 1 ± 0.1 N under different gastrointestinal pH conditions (2.0, 4.0, 6.0 and 7.0). The triborheological studies showed that, independently of the pH conditions, reconstituted mucus exhibited minimal variations in its lubricant properties under low velocities. However, when mucus reached the hydrodynamic lubrication regime, the sample with pH 2 exhibited a significant increase in its coefficient of friction (CoF) in comparison with the other mucus samples, as the Stribeck curves of Figure 3 show. This performance is closely related to the isoelectric point of mucus that is found in the range of pH 2.0–3.0 and represents a critical point in which mucin undergoes a charge shift and conformational changes that directly affect its lubrication properties by reducing molecular hydration [43,52].

The reconstituted mucus reached the boundary lubrication stage with CoF values around 0.16 ± 0.017 in the angular velocity range of 0.01 to 0.6 rad/s, followed by a rapid decrease in CoF values to 0.04 ± 0.008 during the mixed lubrication, which was found at the angular velocity range of 0.6 to 3.9 rad/s. Mucus tends to exhibit good lubricant performance in the boundary and mixed lubrication regimes with very low CoF values (around 0.01) because, during the application of shear forces, the friction is reduced because the energy is dissipated by the movements of the water molecules found in the mucus layer [53]. However, the variations in the negative charge density of the mucus through the different pH values studied did not show an increase in the lubricating properties, suggesting that in the boundary and mixed regimes, lubrication depends mainly on other factors [54]. Moreover, the lubricity of reconstituted mucus tends to be very poor (mainly in the boundary stage) as a result of the deglycosylation that mucin undergoes during mucus purification methodologies, affecting mucin hydration at the molecular scale and, consequently, its lubricant properties [4].

Finally, reconstituted mucus reached the hydrodynamic lubrication, in which the pH represented an important variable. Mucus showed a dramatic increase up to 0.26 ± 0.027 in the CoF under highly acidic conditions (pH = 2). In contrast, the samples with slightly acidic (pH = 4 and 6) and neutral (pH = 7) pH exhibited more controlled behavior of their CoF values with an increase to 0.23 ± 0.036, 0.23 ± 0.038 and 0.19 ± 0.031, respectively. This performance is mainly attributed to the pH-dependent viscoelastic behavior of mucus because the higher viscosity of mucus under more neutral conditions allows the formation of fluid films at lower angular velocities, which, in conjunction with the shear thinning property, reduces friction as a result of a hydrodynamic drag at high angular speeds [52,55].

### 3.2. Chitosan:TPP Nanoparticles Preparation and Characterization

Chitosan:TPP nanoparticles were successfully synthetized by ionic gelation methodology. In the first instance, the formation of chitosan:TPP nanoparticles was determined qualitatively by observing a transition in the coloration of the chitosan solution, which changed from a clear solution to a light-blue one, producing the typical opalescence of colloidal particles in suspension due to the Tyndal effect, during the dropwise addition of TPP solution [56,57]. This color change during the coprecipitation is produced by the attraction of TPP phosphates to chitosan amino groups to form ionically crosslinked polymeric nanoparticles [58]. 

Moreover, the sonication step after the ionic gelation method represented a critical stage during the synthesis since the ultrasonic waves in the range reduce particle size and polydispersity index (PDI) of the nanoparticles by crushing the aggregates formed during the synthesis [59,60]. In this case, a frequency of 40 kHz was applied for 20 min resulting in a significant reduction in the nanoparticle size and PDI. 

On the other hand, the wide variation in pH throughout the gastrointestinal tract represents a significant challenge for all the molecules that interact with this hostile environment that ranges from pH 1–3 in the stomach to pH 6–7.5 in the intestine (duodenum, jejunum and ileum) [61]. Consequently, the chitosan:TPP nanoparticles were subjected to different pH conditions (2.0, 4.0, 6.0 and 7.0) in order to analyze how these environmental circumstances affect their particle size, polydispersity index (PDI) and zeta potential. 

#### 3.2.1. Particle Size, Polydispersity Index and Zeta Potential Measurements

The synthesis of chitosan:TPP nanoparticles is achieved through chitosan’s ability to gel spontaneously on contact with polyanionic TPP solution as a result of inter- and intra-molecular cross-linking interaction among chitosan cationic amino groups and polyanionic phosphate groups [62]. Therefore, the mean size of chitosan:TPP nanoparticles is affected by the pH conditions, so their particle size and polydispersity index (PDI) were measured under different pH environments (2.0, 4.0, 6.0 and 7.0), as Table 1 shows.

The size variations exhibited in the results demonstrated that the pH of the medium represents a critical variable involved in the final particle size and PDI of the chitosan:TPP nanoparticles. Chitosan is a weak polyelectrolyte with a pKa value of around 6.5, so its degree of protonation is mainly affected by the pH of the solution; even chitosan is insoluble at neutral and alkaline pH [63]. Under acidic conditions, the primary amino groups of chitosan are protonated (NH3+) by hydrogen ions, acquiring a high-density charge that generates strong repulsion among chitosan molecules and increases the exposition of cationic amino groups that allows greater interaction with the anionic TPP, resulting in the spontaneous formation of small size nanoparticles with a positive charge [64,65]. Instead, when the pH tends to be more neutral (similar to or above the pKa value of chitosan), the positively charged amino groups would be neutralized by undergoing gradual deprotonation, which causes the folding of chitosan molecular chains and, consequently, the potential exposure to TPP anions decreased. Resulting in less crosslinking and larger particle size with reduced positive surface charge, making nanoparticles susceptible to agglomeration [62,66,67].

Otherwise, zeta potential measures the electrostatic charges of nanoparticles, which represents a major factor in phenomena such as dispersion, flocculation or aggregation, and hence zeta potential is associated with the stability of nanoparticle solutions [68]. Since chitosan is considered a pH-sensitive polymer due to its pKa around 6.5, the zeta potential of the nanoparticles exhibited a pH-related dependence, as Table 1 shows. These variations occur as a consequence of changes in the charge density of the nanoparticle surface because chitosan amino groups became protonated at low pH. However, as the pH increases, the zeta potential decreases due to deprotonation of the NH3+ groups, favoring hydrogen bonding and hydrophobic interactions among chitosan chains and hence leading to aggregation [69,70].

#### 3.2.2. Morphological Characterization 91

**Figure 4:**
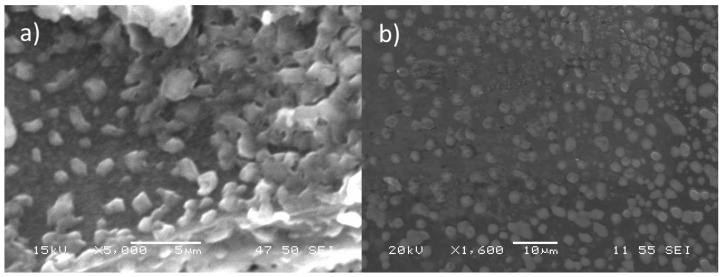
SEM images of (**a**) freeze-dried chitosan:TPP nanoparticles (under high vacuum conditions, applied voltage of 15 kV, magnification of 5000×, working distance 47 mm, spot size 50 nm and secondary electrons detector) and (**b**) Dispersed chitosan:TPP nanoparticles (under high vacuum conditions, applied voltage of 20 kV, magnification of 1600×, working distance 11 mm, spot size 55 nm and secondary electrons detector).

Morphology of the synthetized chitosan:TPP nanoparticles were examined with Scanning Electron Microscope, as Figure 4 shows. The images of the freeze-dried chitosan:TPP nanoparticles (Figure 4a) exhibited a spherical shape, but some particle agglomeration was observed due to the inter- and intramolecular hydrogen bonding produced among -OH groups of chitosan chains during freeze drying procedure [71]. Instead, Figure 4b shows spherical particles well dispersed; as a result, ultrasonic cavitation effects are produced by the 20 min of appropriate conditions of sonication to break down the agglomerates of the resuspended freeze-dried nanoparticles [72]. 

Chitosan:TPP nanoparticles were visualized with a scanning electron microscope to obtain detailed information about their morphology, as Figure 4 shows. The images of the freeze-dried chitosan:TPP nanoparticles (Figure 4a) exhibited a visible particle agglomeration due to the inter- and intramolecular hydrogen bonding produced among -OH groups of chitosan chains during the freeze-drying procedure [71]. Even some of the individual particles seemed to be melting and combining with each other because, during the drying process, the particles severely collapsed [73], giving rise to irregular shapes with a lower value in height relative to the width. This phenomenon commonly occurs in “soft” polymeric particles, such as chitosan, which tend to have a rather loose internal structure [74].

Instead, Figure 4b shows well-dispersed spherical particles with a smooth surface and low polydispersity as a result of ultrasonic cavitation effects produced by the 20 min of appropriated conditions of sonication to break down the agglomerates of the resuspended freeze-dried nanoparticles [72]. Moreover, the nanoparticles in solution exhibited a larger diameter compared to the dry nanoparticles due to the high swelling capacity of chitosan, which stimulates an increase in the size of the nanoparticle to gain stability [75,76].

### 3.3. Assessment of Chitosan Nanoparticles—Mucus Interaction through Rheological Synergism

Mucoadhesion describes the adhesive interaction between the mucus layer and the mucoadhesive polymer due to hydrogen bonding, Van der Waals forces, hydrophobic interactions, ionic interactions and physical entanglement [77,78]. One of the most recommended in vitro approaches to assess mucoadhesion interactions is based on viscosity measurements through rheological synergism [79]. Since the degree of interaction is reflected in the rheological properties of the mixture, for example, high viscosity or viscoelasticity of the mixture mucus–mucoadhesive polymer indicates good mucoadhesiveness [80]. The main advantage of rheology with respect to other methods based on measuring detachment force is the simplicity of evaluating dilute solutions and dispersions [81], such as chitosan:TPP nanoparticles solutions. In this work, rheological measurements were performed to evaluate the mucoadhesive properties of chitosan:TPP nanoparticles under gastrointestinal pH conditions (pH = 2.0, 4.0, 6.0 and 7.0) at body temperature (37 °C).

**Figure 5:**
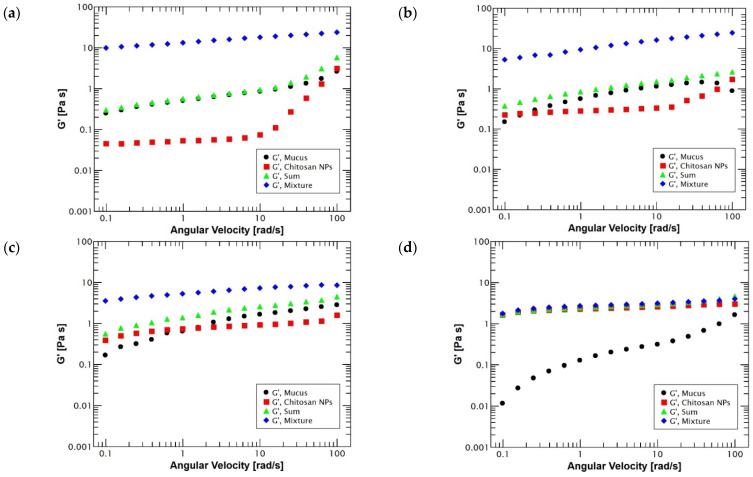
Elastic modulus (*G*′) of reconstituted mucus (black dots); chitosan:TPP nanoparticles (red squares); summation of chitosan:TPP nanoparticles—reconstituted mucus (green triangles); mixture of chitosan:TPP nanoparticles—reconstituted mucus (blue diamonds) for evaluating chitosan:TPP nanoparticles mucoadhesivity through rheological synergism (Δ*G*′) under different pH conditions: (**a**) pH 2, (**b**) pH 4, (**c**) pH 6 and (**d**) pH 7. Mixtures under acidic pH conditions (pH = 2, 4 and 6) exhibited an elastic modulus much larger than summations in a clear performance of a strong polymeric mucoadhesion. Instead, the mixture at neutral pH (pH = 7) exhibited no significant difference with summation in negligible or absent mucoadhesion. N = 5.

The oscillation frequency tests displayed in Figure 5a–c show a positive rheological synergism (Δ*G*′) under acidic conditions (pH = 2, 4 and 6) because the elastic modulus (*G*′) is larger in the mixture of mucin–chitosan:TPP nanoparticles than in the individual and in the sum curves. These results showed a high affinity of chitosan:TPP nanoparticles for reconstituted mucus based on an interaction mainly mediated by the opposite charges of the two biopolymers that influence an entanglement of mucin and polymer chains [82], causing the formation of a resulting polymeric network with an enhanced elastic modulus (*G*′) in comparison with the sum of the elastic modulus (*G*′) values of the single components [83]. Moreover, the rheological synergism results shown in Figure 5d demonstrated that under neutral conditions (pH = 7), the mucoadhesion of chitosan nanoparticles is quite decreased. The values of the rheological synergism and those obtained by the sum of the elastic modulus (*G*′) of the mucus and the nanoparticles are not significantly different (*p* < 0.05). Thus, a negligible mucoadhesion is established as a consequence of the deprotonation of the amino groups in chitosan:TPP nanoparticles at neutral pH, forming an insoluble polymer layer, making difficult the interpenetration among polymeric and mucin chains [78,84]. 

This mucoadhesive profile of chitosan:TPP nanoparticles is mainly attributed to the formation of secondary bonds and electrostatic interactions of positively charged amino groups of chitosan with negatively charged mucus moieties, such as sialic and sulfonic acid residues [85,86]. However, these interactions depend significantly on the protonation state of the functional groups of the polymer, which are closely related to the pH [87]. Consequently, the more acidic the environment is during the interaction, the higher degree of protonation on their surface the chitosan:TPP nanoparticles exhibit, which allows a greater number of interactions with the negatively charged mucosal compounds through the formation of polyelectrolyte complexes that enhance adhesion [88,89], as Figure 5a–c shows. Nevertheless, under neutral or alkaline pH conditions, the amino groups deprotonate, and the positive charges of chitosan are neutralized, forming an insoluble biopolymer layer that tends to precipitate, limiting its mucoadhesive properties [84,90,91], as Figure 5d demonstrate.

**Figure 6:**
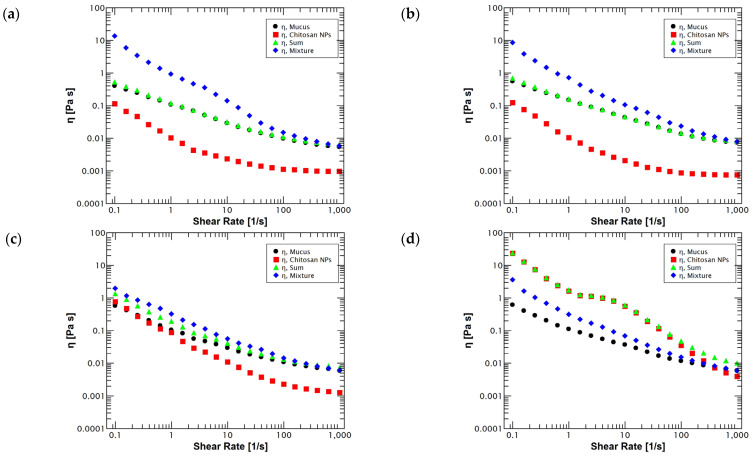
Apparent viscosity (η) of reconstituted mucus (black dots); chitosan:TPP nanoparticles (red squares); summation of chitosan:TPP nanoparticles—reconstituted mucus (green triangles); and mixture of chitosan:TPP nanoparticles—reconstituted mucus (blue diamonds) for evaluating chitosan:TPP nanoparticles mucoadhesivity through rheological synergism (Δ*G*′) under different pH conditions: (**a**) pH 2, (**b**) pH 4, (**c**) pH 6 and (**d**) pH 7. Mixtures under highly acidic pH conditions (pH = 2 and 4) exhibited a larger viscosity than summations in an evident mucoadhesive performance. Mixture at slightly acidic (pH = 6) conditions displayed a minimum mucoadhesive behavior. Moreover, the mixture under a neutral environment (pH = 7) expressed no significant difference with summation in insignificant or null mucoadhesion. N = 5.

Additionally, a viscosity-based synergistic approach can be taken to determine rheological synergism (Δ η) based on the difference between the viscosity of the mixture and the sum of the individual components [87]. The viscosity synergism of a molecular dispersion of a fully hydrated polymer and mucin can be considered a reflection of the mucoadhesive strength of the polymer-mucin system [92] because the chain entanglement and molecular bonding among the mucin and the mucoadhesive polymer alter the rheological behavior of both materials [80]. Therefore, flow sweep tests shown in Figure 6 were performed to corroborate the positive rheological synergism previously described with the oscillation frequency tests. The graphs showed that, independently of the pH conditions, the mixture preserved a non-Newtonian shear thinning behavior that is characteristic of a polymeric solution. 

Furthermore, the results demonstrated that at highly acidic values (pH = 2 and 4), there is a clear presence of a positive rheological synergism due to the viscosity values of the mixture being significantly higher (*p* < 0.05) than the sum of the individual viscosities as Figure 6a,b shows. These strong mucoadhesive interactions are due to the cationic nature of chitosan and the high degree of interpenetration between mucin glycoproteins and chitosan chains, leading to the formation of secondary chemical bonds between both chains, hence, increasing the viscosity of the samples through rheological synergism [93,94]. Instead, as the environmental conditions approach neutral pH values (pH = 6), the rheological synergism value decreases and becomes not significantly different (*p* < 0.05), although it remains positive. These changes are produced by the influence of chitosan ionizable groups that tend to deprotonate near its pKa value (6.5), reducing the charge density in the surface of chitosan:TPP nanoparticles and, consequently, reducing the electrostatic interactions [89,95]. Finally, the tests revealed that samples at neutral pH (pH = 7) exhibited a negative synergism, indicating that the mucoadhesion phenomenon is not present due to chitosan being completely deprotonated, resulting in a serious reduction in its electrostatic charges [96] and possibly in the collapse of the nanoparticles [97].

**Figure 7:**
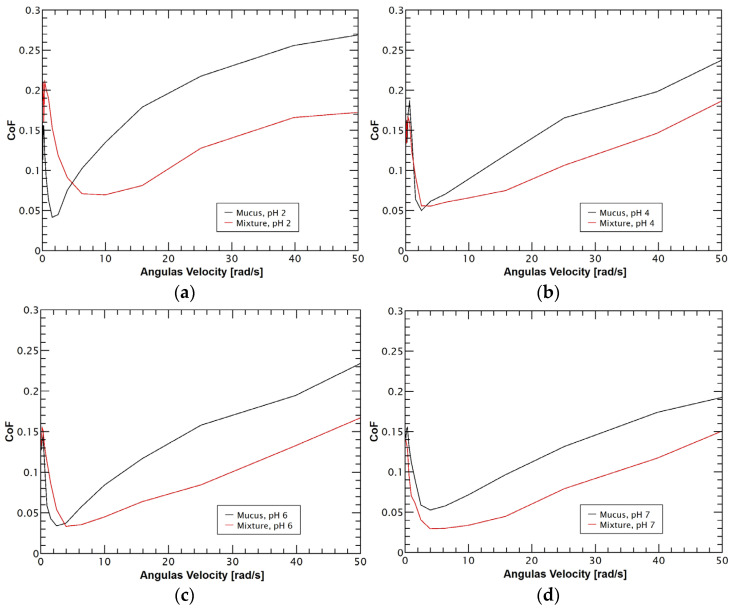
Comparison between the coefficients of friction (CoF) of the reconstituted mucus and the mixture of reconstituted mucus–chitosan:TPP nanoparticles at 37 °C under different pH conditions. (**a**) Stribeck curve at pH 2. (**b**) Stribeck curve at pH 4. (**c**) Stribeck curve at pH 6. (**d**) Stribeck curve at pH 7. All the mixtures exhibited improved lubricant properties regarding mucus samples. N = 3.

In the same way, triborheological testing of the reconstituted mucus–chitosan:TPP system demonstrated that mucoadhesion enhanced the lubricant properties of mucus, as Figure 7 displays through different Stribeck. Mucus–polymeric nanoparticle mixture samples reached the boundary lubrication stage in the same angular velocity range as reconstituted mucus (0.01 to 0.6 rad/s). Followed by the mixed lubrication, which was found at the angular velocity range of 0.6 to 3.9 rad/s, with the exception of the mixture at pH 2 that exhibited an elongation of the mixed lubrication stage up to 9.99 rad/s. Moreover, during the hydrodynamic phase, the mixture under the most acidic conditions showed a greater improvement in its lubricating conditions. The increase in the coefficient of friction exhibited a less pronounced slope in comparison to the other mixtures (Figure 7a–d) and presented a wider reduction regarding reconstituted mucus values, as can be seen in Table 2. 

This significant improvement in the lubricating properties of the mixture under acidic pH conditions was produced due to protonated chitosan acting as a physical crosslinker with mucus through chain entanglement and formation of electrostatic bridges, increasing mucus load bearing and wear resistance capabilities [98]. Instead, under neutral conditions, chitosan exhibits poor solubility and limited electrostatic interactions due to its negligible charge, resulting in a significant reduction in the formation of mucin-polymer aggregates [99,100]. Therefore, the synergetic lubricity is mainly produced by the establishment of hydrogen bonds, and hence, the synergetic lubricant effects among mucus and the polymeric nanoparticles are diminished [101]. 

## 4. Conclusions

Triborheological evaluation of reconstituted mucus confirmed the pH dependence of its viscoelastic properties. Under extremely acidic conditions, reconstituted mucus acquires an elastic “weak gel” behavior. In contrast, under neutral conditions, mucus acts as a polymeric solution with lubricating properties. Additionally, triborheology showed that reconstituted mucus viscosity preserved its non-Newtonian shear thinning behavior independently of the pH conditions. However, the lubricating properties of the reconstituted mucus were limited, mainly in acid conditions.

Chitosan:TPP nanoparticles proved to be sensitive to pH variations. Nanoparticles exhibited a small size (171.01 ± 21.86 nm) and highly positive zeta potential (23.55 ± 0.84 mV) in an extremely acidic environment. In contrast, the size of the nanoparticles increased up to 506.23 ± 49.98 nm with a zeta potential close to zero (3.87 ± 0.79 nm) at neutral conditions. This performance is related to the cationic nature of chitosan (pKa∼6.5) and the protonation/deprotonation of its amino groups. 

The mucoadhesive profile of the chitosan:TPP nanoparticles was evaluated by rheological synergism with a *p*-value > 0.05. The elastic modulus term (Δ*G*′) showed a positive synergism for all the samples under acidic conditions (pH = 2, 4 and 6), while the sample at neutral conditions (pH = 7) exhibited a negligible synergism. Instead, the viscosity (η) synergism was only observed in highly acidic conditions (pH = 2 and 4), whereas slightly acidic (pH = 6) and neutral (pH = 7) samples showed imperceptible synergism. Considering that both rheological synergism studies are complementary, the mucoadherence phenomenon only occurs at acidic pH since the amino groups of chitosan are protonated and establish electrostatic interactions with negatively charged mucus. 

Finally, the Stribeck curves of the mixtures of reconstituted mucus–chitosan:TPP nanoparticles proved that mucoadhesion is related to enhanced lubricant properties because the physical chain entanglement and formation of electrostatic interactions among mucin and chitosan molecules are associated with better load-bearing and wear resistance properties. 

## Figures and Tables

**Table 1:** Average particle size, polydispersity index (PDI) and zeta potential of chitosan:TPP nanoparticles under different pH conditions. N = 3.

pH	Size (nm)	PDI	Zeta Potential (mV)
2.0	171.01 ± 21.86	0.29 ± 0.03	23.55 ± 0.84
4.0	297.53 ± 31.95	0.41 ± 0.11	19.69 ± 0.21
6.0	350.03 ± 38.84	0.42 ± 0.06	16.23 ± 1.95
7.0 ^1^	506.23 ± 49.98	0.61 ± 0.04	3.87 ± 0.79

^1^ pH value above chitosan pKa (~6.5).

**Table 2:** Comparison of the coefficient of friction values between reconstituted mucus alone and reconstituted mucus–chitosan:TPP nanoparticles mixture during all the lubrication regimes (boundary lubrication, mixed lubrication and hydrodynamic lubrication) under different pH conditions. N = 3.

	Boundary Lubrication		Mixed Lubrication		Hydrodynamic Lubrication	
pH	Mucus	Mixture	Mucus	Mixture	Mucus	Mixture
2.0	0.15 ± 0.035	0.21 ± 0.081	0.04 ± 0.019	0.07 ± 0.006	0.27 ± 0.026	0.17 ± 0.024
4.0	0.19 ± 0.029	0.17 ± 0.037	0.05 ± 0.016	0.06 ± 0.031	0.24 ± 0.044	0.19 ± 0.008
6.0	0.14 ± 0.028	0.13 ± 0.049	0.03 ± 0.008	0.03 ± 0.012	0.23 ± 0.054	0.17 ± 0.030
7.0 ^1^	0.16 ± 0.028	0.14 ± 0.008	0.05 ± 0.041	0.03 ± 0.003	0.19 ± 0.016	0.15 ± 0.049

^1^ pH value above chitosan pKa (~6.5).

## Data Availability

Not applicable.

## References

[1] Griffiths P.C., Cattoz B., Ibrahim M.S., Anuonye J.C. (2015). Probing the interaction of nanoparticles with mucin for drug delivery applications using dynamic light scattering. Eur. J. Pharm. Biopharm..

[2] Li L.D., Crouzier T., Sarkar A., Dunphy L., Han J., Ribbeck K. (2013). Spatial Configuration and Composition of Charge Modulates Transport into a Mucin Hydrogel Barrier. Biophys. J..

[3] Wilcox M., Van Rooij L., Chater P., de Sousa I.P., Pearson J. (2015). The effect of nanoparticle permeation on the bulk rheological properties of mucus from the small intestine. Eur. J. Pharm. Biopharm..

[4] Crouzier T., Boettcher K., Geonnotti A.R., Kavanaugh N.L., Hirsch J.B., Ribbeck K., Lieleg O. (2015). Modulating Mucin Hydration and Lubrication by Deglycosylation and Polyethylene Glycol Binding. Adv. Mater. Interfaces.

[5] Bansil R., Turner B.S. (2018). The biology of mucus: Composition, synthesis and organization. Adv. Drug Deliv. Rev..

[6] Quintana-Hayashi M.P., Padra M., Padra J.T., Benktander J., Lindén S.K. (2018). Mucus-Pathogen Interactions in the Gastrointestinal Tract of Farmed Animals. Microorganisms.

[7] Ahn J., Crouzier T., Ribbeck K., Rubner M.F., Cohen R.E. (2014). Tuning the Properties of Mucin via Layer-by-Layer Assembly. Biomacromolecules.

[8] Leal J., Smyth H.D., Ghosh D. (2017). Physicochemical properties of mucus and their impact on transmucosal drug delivery. Int. J. Pharm..

[9] Carlson T.L., Lock J.Y., Carrier R.L. (2018). Engineering the Mucus Barrier. Annu. Rev. Biomed. Eng..

[10] Yang L., Wu X., Luo M., Shi T., Gong F., Yan L., Li J., Ma T., Li R., Liu H. (2022). Na^+^/Ca^2+^ induced the migration of soy hull polysaccharides in the mucus layer in vitro. Int. J. Biol. Macromol..

[11] Ruiz-Pulido G., Medina D.I. (2020). An overview of gastrointestinal mucus rheology under different pH conditions and introduction to pH-dependent rheological interactions with PLGA and chitosan nanoparticles. Eur. J. Pharm. Biopharm..

[12] Rajput G., Majmudar F., Patel J., Thakor R., Rajgor N. (2010). Stomach-specific mucoadhesive microsphere as a controlled drug delivery system. Syst. Rev. Pharm..

[13] Pool H., Quintanar D., Figueroa J.D.D., Bechara J.E.H., McClements D.J., Mendoza S. (2012). Polymeric Nanoparticles as Oral Delivery Systems for Encapsulation and Release of Polyphenolic Compounds: Impact on Quercetin Antioxidant Activity & Bioaccessibility. Food Biophys..

[14] Lin W., Kluzek M., Iuster N., Shimoni E., Kampf N., Goldberg R., Klein J. (2020). Cartilage-inspired, lipid-based boundary-lubricated hydrogels. Science.

[15] Zhu J., Zhang X., Qin Z., Zhang L., Ye Y., Cao M., Gao L., Jiao T. (2020). Preparation of PdNPs doped chitosan-based composite hydrogels as highly efficient catalysts for reduction of 4-nitrophenol. Colloids Surf. A Physicochem. Eng. Asp..

[16] Mamidi N., Delgadillo R.M.V., González-Ortiz A. (2020). Engineering of carbon nano-onion bioconjugates for biomedical applications. Mater. Sci. Eng. C.

[17] Qian Q., Shi L., Gao X., Ma Y., Yang J., Zhang Z., Qian J., Zhu X. (2019). A Paclitaxel-Based Mucoadhesive Nanogel with Multivalent Interactions for Cervical Cancer Therapy. Small.

[18] Leyva-Gómez G., Piñón-Segundo E., Mendoza-Muñoz N., Zambrano-Zaragoza M.L., Mendoza-Elvira S., Quintanar-Guerrero D. (2018). Approaches in Polymeric Nanoparticles for Vaginal Drug Delivery: A Review of the State of the Art. Int. J. Mol. Sci..

[19] Danhier F., Ansorena E., Silva J.M., Coco R., Le Breton A., Préat V. (2012). PLGA-based nanoparticles: An overview of biomedical applications. J. Control. Release.

[20] McNamara K., Tofail S.A.M. (2016). Nanoparticles in biomedical applications. Adv. Phys. X.

[21] Mitragotri S., Burke P.A., Langer R. (2014). Overcoming the challenges in administering biopharmaceuticals: Formulation and delivery strategies. Nat. Rev. Drug Discov..

[22] Ruiz-Pulido G., Medina D., Barani M., Rahdar A., Sargazi G., Baino F., Pandey S. (2021). Nanomaterials for the Diagnosis and Treatment of Head and Neck Cancers: A Review. Materials.

[23] Garg U., Chauhan S., Nagaich U., Jain N. (2019). Current Advances in Chitosan Nanoparticles Based Drug Delivery and Targeting. Adv. Pharm. Bull..

[24] Hembram K.C., Prabha S., Chandra R., Ahmed B., Nimesh S. (2014). Advances in preparation and characterization of chitosan nanoparticles for therapeutics. Artif. Cells Nanomed. Biotechnol..

[25] Mohammed M.A., Syeda J.T.M., Wasan K.M., Wasan E.K. (2017). An Overview of Chitosan Nanoparticles and Its Application in Non-Parenteral Drug Delivery. Pharmaceutics.

[26] Shim S., Yoo H. (2020). The Application of Mucoadhesive Chitosan Nanoparticles in Nasal Drug Delivery. Mar. Drugs.

[27] Pola C.C., Moraes A.R., Medeiros E.A., Teófilo R.F., Soares N.F., Gomes C.L. (2019). Development and optimization of pH-responsive PLGA-chitosan nanoparticles for triggered release of antimicrobials. Food Chem..

[28] Sun L., Nie X., Lu W., Zhang Q., Fang W., Gao S., Chen S., Hu R. (2022). Mucus-Penetrating Alginate-Chitosan Nanoparticles Loaded with Berberine Hydrochloride for Oral Delivery to the Inflammation Site of Ulcerative Colitis. AAPS PharmSciTech.

[29] Dyawanapelly S., Koli U., Dharamdasani V., Jain R., Dandekar P. (2016). Improved mucoadhesion and cell uptake of chitosan and chitosan oligosaccharide surface-modified polymer nanoparticles for mucosal delivery of proteins. Drug Deliv. Transl. Res..

[30] Choi C., Kim S., Pak P., Yoo D., Chung Y. (2007). Effect of N-acylation on structure and properties of chitosan fibers. Carbohydr. Polym..

[31] Guo Z., Chen R., Xing R., Liu S., Yu H., Wang P., Li C., Li P. (2006). Novel derivatives of chitosan and their antifungal activities in vitro. Carbohydr. Res..

[32] Kazachenko A.S., Akman F., Malyar Y.N., Issaoui N., Vasilieva N.Y., Karacharov A.A. (2021). Synthesis optimization, DFT and physicochemical study of chitosan sulfates. J. Mol. Struct..

[33] Zhao D., Yu S., Sun B., Gao S., Guo S., Zhao K. (2018). Biomedical Applications of Chitosan and Its Derivative Nanoparticles. Polymers.

[34] Mansuri S., Kesharwani P., Jain K., Tekade R.K., Jain N.K. (2016). Mucoadhesion: A promising approach in drug delivery system. React. Funct. Polym..

[35] Grießinger J., Dünnhaupt S., Cattoz B., Griffiths P., Oh S., i Gómez S.B., Wilcox M., Pearson J., Gumbleton M., Abdulkarim M. (2015). Methods to determine the interactions of micro- and nanoparticles with mucus. Eur. J. Pharm. Biopharm..

[36] Baus R.A., Zahir-Jouzdani F., Dünnhaupt S., Atyabi F., Bernkop-Schnürch A. (2019). Mucoadhesive hydrogels for buccal drug delivery: In vitro-in vivo correlation study. Eur. J. Pharm. Biopharm..

[37] Madsen F., Eberth K., Smart J.D. (1998). A rheological assessment of the nature of interactions between mucoadhesive polymers and a homogenised mucus gel. Biomaterials.

[38] Riley R.G., Smart J.D., Tsibouklis J., Dettmar P.W., Hampson F., Davis J., Kelly G., Wilber W.R. (2001). An investigation of mucus/polymer rheological synergism using synthesised and characterised poly(acrylic acid)s. Int. J. Pharm..

[39] Demouveaux B., Gouyer V., Gottrand F., Narita T., Desseyn J.-L. (2018). Gel-forming mucin interactome drives mucus viscoelasticity. Adv. Colloid Interface Sci..

[40] de Oliveira Cardoso V.M., Gremião M.P.D., Cury B.S.F. (2020). Mucin-polysaccharide interactions: A rheological approach to evaluate the effect of pH on the mucoadhesive properties. Int. J. Biol. Macromol..

[41] Collado-González M., Espinosa Y.G., Goycoolea F.M. (2019). Interaction Between Chitosan and Mucin: Fundamentals and Applications. Biomimetics.

[42] Celli J.P., Turner B.S., Afdhal N.H., Ewoldt R.H., McKinley G.H., Bansil R., Erramilli S. (2007). Rheology of Gastric Mucin Exhibits a pH-Dependent Sol–Gel Transition. Biomacromolecules.

[43] Caicedo J.A., Perilla J.E. (2015). Effect of pH on the rheological response of reconstituted gastric mucin. Ing. Investig..

[44] Ahmad M., Ritzoulis C., Chen J. (2018). Shear and extensional rheological characterisation of mucin solutions. Colloids Surf. B Biointerfaces.

[45] Duque-Ossa L., Ruiz-Pulido G., Medina D. (2021). Triborheological Study under Physiological Conditions of PVA Hydrogel/HA Lubricant as Synthetic System for Soft Tissue Replacement. Polymers.

[46] Georgiades P., Pudney P.D.A., Thornton D.J., Waigh T.A. (2014). Particle tracking microrheology of purified gastrointestinal mucins. Biopolymers.

[47] Menchicchi B., Fuenzalida J.P., Hensel A., Swamy M.J., David L., Rochas C., Goycoolea F.M. (2015). Biophysical Analysis of the Molecular Interactions between Polysaccharides and Mucin. Biomacromolecules.

[48] Abodinar A., Tømmeraas K., Ronander E., Smith A., Morris G.A. (2016). The physicochemical characterisation of pepsin degraded pig gastric mucin. Int. J. Biol. Macromol..

[49] Khutoryanskiy V.V. (2010). Advances in Mucoadhesion and Mucoadhesive Polymers. Macromol. Biosci..

[50] Boegh M., Nielsen H.M. (2014). Mucus as a Barrier to Drug Delivery—Understanding and Mimicking the Barrier Properties. Basic Clin. Pharmacol. Toxicol..

[51] Ensign L.M., Cone R., Hanes J. (2012). Oral drug delivery with polymeric nanoparticles: The gastrointestinal mucus barriers. Adv. Drug Deliv. Rev..

[52] Coles J.M., Chang D.P., Zauscher S. (2010). Molecular mechanisms of aqueous boundary lubrication by mucinous glycoproteins. Curr. Opin. Colloid Interface Sci..

[53] Schömig V.J., Käsdorf B.T., Scholz C., Bidmon K., Lieleg O., Berensmeier S. (2016). An optimized purification process for porcine gastric mucin with preservation of its native functional properties. RSC Adv..

[54] Yakubov G.E., McColl J., Bongaerts J.H.H., Ramsden J.J. (2009). Viscous Boundary Lubrication of Hydrophobic Surfaces by Mucin. Langmuir.

[55] Bongaerts J., Fourtouni K., Stokes J. (2007). Soft-tribology: Lubrication in a compliant PDMS–PDMS contact. Tribol. Int..

[56] Liu P., Zhao X. (2013). Facile preparation of well-defined near-monodisperse chitosan/sodium alginate polyelectrolyte complex nanoparticles (CS/SAL NPs) via ionotropic gelification: A suitable technique for drug delivery systems. Biotechnol. J..

[57] Vila-Sanjurjo C., David L., Remuñán-López C., Goycoolea F. (2019). Effect of the ultrastructure of chitosan nanoparticles in colloidal stability, quorum quenching and antibacterial activities. J. Colloid Interface Sci..

[58] Alishahi A., Mirvaghefi A., Tehrani M., Farahmand H., Shojaosadati S.A., Dorkoosh F., Elsabee M.Z. (2011). Shelf life and delivery enhancement of vitamin C using chitosan nanoparticles. Food Chem..

[59] Büyük N.Ï., Arayici P.P., Derman S., Mustafaeva Z., Yücel S. (2020). An Optimization Study for Chitosan Nanoparticles: Synthesis and Characterization. Celal Bayar Univ. J. Sci..

[60] Tang E., Huang M., Lim L.Y. (2003). Ultrasonication of chitosan and chitosan nanoparticles. Int. J. Pharm..

[61] Barbosa A.I., Costa Lima S.A., Reis S. (2019). Application of pH-Responsive Fucoidan/Chitosan Nanoparticles to Improve Oral Quercetin Delivery. Molecules.

[62] Makhlof A., Tozuka Y., Takeuchi H. (2011). Design and evaluation of novel pH-sensitive chitosan nanoparticles for oral insulin delivery. Eur. J. Pharm. Sci..

[63] Fan W., Yan W., Xu Z., Ni H. (2012). Formation mechanism of monodisperse, low molecular weight chitosan nanoparticles by ionic gelation technique. Colloids Surf. B Biointerfaces.

[64] Dudhani A.R., Kosaraju S.L. (2010). Bioadhesive chitosan nanoparticles: Preparation and characterization. Carbohydr. Polym..

[65] Sullivan D.J., Cruz-Romero M., Collins T., Cummins E., Kerry J.P., Morris M.A. (2018). Synthesis of monodisperse chitosan nanoparticles. Food Hydrocoll..

[66] Mattu C., Li R., Ciardelli G. (2013). Chitosan nanoparticles as therapeutic protein nanocarriers: The effect of ph on particle formation and encapsulation efficiency. Polym. Compos..

[67] Tang Z.-X., Qian J.-Q., Shi L.-E. (2007). Preparation of chitosan nanoparticles as carrier for immobilized enzyme. Appl. Biochem. Biotechnol..

[68] Hadidi M., Pouramin S., Adinepour F., Haghani S., Jafari S.M. (2020). Chitosan nanoparticles loaded with clove essential oil: Characterization, antioxidant and antibacterial activities. Carbohydr. Polym..

[69] de Moura M.R., Aouada M.R.D.M., Mattoso L.H. (2008). Preparation of chitosan nanoparticles using methacrylic acid. J. Colloid Interface Sci..

[70] Athavale R., Sapre N., Rale V., Tongaonkar S., Manna G., Kulkarni A., Shirolkar M.M. (2021). Tuning the surface charge properties of chitosan nanoparticles. Mater. Lett..

[71] Thandapani G., Supriya P., Sudha P.N., Sukumaran A. (2017). Size optimization and in vitro biocompatibility studies of chitosan nanoparticles. Int. J. Biol. Macromol..

[72] Gokce Y., Cengiz B., Yildiz N., Calimli A., Aktas Z. (2014). Ultrasonication of chitosan nanoparticle suspension: Influence on particle size. Colloids Surfaces A Physicochem. Eng. Asp..

[73] Ding Z., Mo M., Zhang K., Bi Y., Kong F. (2021). Preparation, characterization and biological activity of proanthocyanidin-chitosan nanoparticles. Int. J. Biol. Macromol..

[74] Liu H., Chen B., Mao Z., Gao C. (2007). Chitosan nanoparticles for loading of toothpaste actives and adhesion on tooth analogs. J. Appl. Polym. Sci..

[75] Kahdestani S.A., Shahriari M.H., Abdouss M. (2020). Synthesis and characterization of chitosan nanoparticles containing teicoplanin using sol–gel. Polym. Bull..

[76] Mukhopadhyay P., Sarkar K., Chakraborty M., Bhattacharya S., Mishra R., Kundu P. (2013). Oral insulin delivery by self-assembled chitosan nanoparticles: In vitro and in vivo studies in diabetic animal model. Mater. Sci. Eng. C.

[77] García-Guzmán P., Medina-Torres L., Calderas F., Bernad-Bernad M., Gracia-Mora J., Marcos X., Correa-Basurto J., Núñez-Ramírez D., Manero O. (2019). Rheological mucoadhesion and cytotoxicity of montmorillonite clay mineral/hybrid microparticles biocomposite. Appl. Clay Sci..

[78] da Silva J.B., dos Santos R.S., da Silva M.B., Braga G., Cook M.T., Bruschi M.L. (2020). Interaction between mucoadhesive cellulose derivatives and Pluronic F127: Investigation on the micelle structure and mucoadhesive performance. Mater. Sci. Eng. C.

[79] Silva M.M., Calado R., Marto J., Bettencourt A., Almeida A.J., Gonçalves L.M.D. (2017). Chitosan Nanoparticles as a Mucoadhesive Drug Delivery System for Ocular Administration. Mar. Drugs.

[80] Dodou D., Breedveld P., Wieringa P.A. (2005). Mucoadhesives in the gastrointestinal tract: Revisiting the literature for novel applications. Eur. J. Pharm. Biopharm..

[81] Bonferoni M., Sandri G., Ferrari F., Rossi S., Larghi V., Zambito Y., Caramella C. (2010). Comparison of different in vitro and ex vivo methods to evaluate mucoadhesion of glycol-palmitoyl chitosan micelles. J. Drug Deliv. Sci. Technol..

[82] Rossi S., Vigani B., Bonferoni M.C., Sandri G., Caramella C., Ferrari F. (2018). Rheological analysis and mucoadhesion: A 30 year-old and still active combination. J. Pharm. Biomed. Anal..

[83] Jelkmann M., Leichner C., Menzel C., Kreb V., Bernkop-Schnürch A. (2019). Cationic starch derivatives as mucoadhesive and soluble excipients in drug delivery. Int. J. Pharm..

[84] Lu B., Lv X., Le Y. (2019). Chitosan-Modified PLGA Nanoparticles for Control-Released Drug Delivery. Polymers.

[85] Bernkop-Schnürch A., Weithaler A., Albrecht K., Greimel A. (2006). Thiomers: Preparation and in vitro evaluation of a mucoadhesive nanoparticulate drug delivery system. Int. J. Pharm..

[86] Raj P.M., Raj R., Kaul A., Mishra A.K., Ram A. (2018). Biodistribution and targeting potential assessment of mucoadhesive chitosan nanoparticles designed for ulcerative colitis via scintigraphy. RSC Adv..

[87] Dave R.S., Goostrey T.C., Ziolkowska M., Czerny-Holownia S., Hoare T., Sheardown H. (2021). Ocular drug delivery to the anterior segment using nanocarriers: A mucoadhesive/mucopenetrative perspective. J. Control. Release.

[88] Lee C.-A., Kim B.-S., Cho C.-W. (2016). Quantitative evaluation of mucoadhesive polymers to compare the mucoadhesion. J. Pharm. Investig..

[89] Coutinho A.J., Lima S., Afonso C., Reis S. (2020). Mucoadhesive and pH responsive fucoidan-chitosan nanoparticles for the oral delivery of methotrexate. Int. J. Biol. Macromol..

[90] Mukhopadhyay P., Mishra R., Rana D., Kundu P.P. (2012). Strategies for effective oral insulin delivery with modified chitosan nanoparticles: A review. Prog. Polym. Sci..

[91] Ways T.M.M., Lau W.M., Khutoryanskiy V.V. (2018). Chitosan and Its Derivatives for Application in Mucoadhesive Drug Delivery Systems. Polymers.

[92] Thirawong N., Kennedy R.A., Sriamornsak P. (2008). Viscometric study of pectin–mucin interaction and its mucoadhesive bond strength. Carbohydr. Polym..

[93] Hajikhani M., Emam-Djomeh Z., Jafari S.M. (2020). Chapter Eleven—Mucoadhesive delivery systems for nanoencapsulated food ingredients. Nanoencapsulation in the Food Industry.

[94] Sahatsapan N., Rojanarata T., Ngawhirunpat T., Opanasopit P., Tonglairoum P. (2018). 6-Maleimidohexanoic acid-grafted chitosan: A new generation mucoadhesive polymer. Carbohydr. Polym..

[95] Donnelly R.F., Shaikh R., Singh R.R.T., Garland M.J., Woolfson A.D. (2011). Mucoadhesive drug delivery systems. J. Pharm. Bioallied Sci..

[96] Chen C.-H., Lin Y.-S., Wu S.-J., Mi F.-L. (2018). Mutlifunctional nanoparticles prepared from arginine-modified chitosan and thiolated fucoidan for oral delivery of hydrophobic and hydrophilic drugs. Carbohydr. Polym..

[97] Lin Y.-H., Chang C.-H., Wu Y.-S., Hsu Y.-M., Chiou S.-F., Chen Y.-J. (2009). Development of pH-responsive chitosan/heparin nanoparticles for stomach-specific anti-Helicobacter pylori therapy. Biomaterials.

[98] Nikogeorgos N., Efler P., Kayitmazer A.B., Lee S. (2014). “Bio-glues” to enhance slipperiness of mucins: Improved lubricity and wear resistance of porcine gastric mucin (PGM) layers assisted by mucoadhesion with chitosan. Soft Matter.

[99] Nikogeorgos N., Patil N.J., Zappone B., Lee S. (2016). Interaction of porcine gastric mucin with various polycations and its influence on the boundary lubrication properties. Polymer.

[100] Ahmad M., Ritzoulis C., Pan W., Chen J. (2020). Biologically-relevant interactions, phase separations and thermodynamics of chitosan–mucin binary systems. Process Biochem..

[101] Szilágyi B., Mammadova A., Gyarmati B., Szilágyi A. (2020). Mucoadhesive interactions between synthetic polyaspartamides and porcine gastric mucin on the colloid size scale. Colloids Surf. B Biointerfaces.

